# Dandelion Polysaccharide Exerts Anti-Angiogenesis Effect on Hepatocellular Carcinoma by Regulating VEGF/HIF-1α Expression

**DOI:** 10.3389/fphar.2020.00460

**Published:** 2020-04-08

**Authors:** Feng Ren, Kaixuan Wu, Yun Yang, Yingying Yang, Yuxia Wang, Jian Li

**Affiliations:** ^1^School of Basic Medical Sciences, Xinxiang Medical University, Xinxiang, China; ^2^School of Nursing, Xinxiang Medical University, Xinxiang, China; ^3^School of Forensic Medicine, Xinxiang Medical University, Xinxiang, China

**Keywords:** hepatocellular carcinoma, dandelion polysaccharide, angiogenesis, vascular endothelial growth factor, hypoxia-inducible factor 1α

## Abstract

Recent studies have revealed that natural plants-derived polysaccharides exhibit potent anti-tumor activity. Our earlier studies suggest that dandelion polysaccharide (DP) inhibits hepatocellular carcinoma (HCC) cell proliferation *in vitro* and *in vivo*. Here, we investigated the effects of DP on the angiogenesis of HCC and the potential molecular mechanisms by which DP regulates angiogenesis. Wound-healing and transwell invasion assays revealed that DP inhibited HUVECs migration and invasion *in vitro*, respectively. Tube formation assay, chick chorioallantoic membrane (CAM) assay, and immunohistochemistry (IHC) demonstrated that DP suppressed vasculogenesis *in vitro* and *in vivo*. Moreover, Western blot and immunofluorescence staining verified that DP treatment decreased the protein levels of some key factors involved in angiogenesis of HCC, such as hypoxia-inducible factor 1α (HIF-1α), vascular endothelial growth factor (VEGF), p-PI3K, and p-AKT. However, activation of PI3K/AKT pathway with insulin-like growth factor 1 (IGF-1) treatment attenuated the effect of DP on angiogenesis *via* lowering the expression of HIF-1α and VEGF. In summary, we found that DP treatment inhibited angiogenesis *in vivo* and *in vitro* through suppressing expression of VEGF and HIF-1a. Furthermore, we showed that the expression of VEGF and HIF1-α was modulated by PI3K/AKT signaling. Collectively, our study suggests that DP is a promising anti-cancer drug candidate for treating HCC.

## Introduction

Hepatocellular carcinoma (HCC) is the sixth most common malignancy worldwide and ranks the fourth cause of lethal tumor (Bray et al., 2018). HCC is a typical hypervascular tumor and commonly associated with hypervascularity (Srivastava et al., 2018; Morse et al., 2019). Therefore, inhibiting angiogenesis is believed to be a potential strategy to control HCC (Taketomi, 2016; Barbhuiya et al., 2017).

Current approaches for HCC therapy include surgical resection, chemotherapy, and liver transplantation. Surgical resection is the most widely used and effective treatment for HCC. As most patients are often diagnosed at the intermediate-advanced stage and unable to surgical therapies, chemotherapy is still one of the essential means in the treatment of HCC. However, the drug resistance and severe toxic side effects of chemotherapy are the important factors to limitation the clinical application of chemotherapy. Clinical practices have improved that Chinese medicine definitely improve the effect of chemotherapy and reduce adverse effect. Thus, finding Chinese medicine with therapeutic effects and less toxicities for the prevention and treatment of HCC are urgently needed.

Vascular endothelial growth factor (VEGF), one of the most prominent regulators involving vasculogenesis, is highly expressed in human HCC specimens (Choi et al., 2017; Liu et al., 2017). Hypoxia is one of the most potent stimuli for increased VEGF production in tumor cells (Liu et al., 2017; Campbell et al., 2019). With tumor tissues growing, one of the critical mediators of hypoxic responses is the hypoxia-inducible factor 1α (HIF-1α). Increasing evidence have demonstrated that the activation of PI3K/AKT signaling pathway elevates VEGF expression by up-regulating HIF-1α (Lee et al., 2015; Blagotinsek and Rozman, 2017; Kim et al., 2017). Thus, identification of drugs that inhibit PI3K/AKT signaling pathway and decrease VEGF and HIF-1α expression is instructive for treating HCC.

Many natural products have been found to possess many bioactivities, such as anti-tumor effects, anti-oxidant function, and anti-inflammatory activity (Newman and Cragg, 2012; Chen et al., 2016a; Chen et al., 2016b; Ren et al., 2018). Dandelion is a popular Chinese medicinal herb widely grown in lawns, roadsides as well as shores of water ways and often consumed as a vegetable. Generally, dandelion is regarded as a medicinal nontoxic herb to treat digestive diseases as well as disorders of the breast, liver, and gallbladder (Jeon et al., 2008; Domitrovic et al., 2010; Oh et al., 2015). Several studies have shown that dandelion possesses anti-carcinogenic activity on breast cancer, lung cancer, and intestinal carcinoma (Mingarro et al., 2015; Oh et al., 2015; Chien et al., 2018; Ding and Wen, 2018). Dandelion polysaccharide (DP), α-type polysaccharides, is derived from the root of dandelion and consisted of glucose, galactose, arabinose, arabinose rhamnose, and glucuronic acid (Chen et al., 2016b; Cai et al., 2017). Our previous findings have demonstrated that DP markedly inhibited the proliferation of HCC cancer cells *in vitro* and *in vivo* and induced cell apoptosis and arrested cell cycle at the G0/G1 phase. Furthermore, *in vivo* studies demonstrated that 400mg/kg DP treatment was well-tolerated by mouse without any adverse systemic toxicological changes (Ren et al., 2019). In the present study, we found that DP inhibited angiogenesis both *in vitro* and *in vivo* through suppression of HIF-1a and VEGF expression which was regulated by PI3K/AKT signaling.

## Materials and Methods

### Cell Lines and Cell Culture

Human HCC cell lines (HepG2) were provided by the Stem Cell Bank, Chinese Academy of Sciences. Mouse HCC cell lines (Hepa1-6 and H22) and Human umbilical vein endothelial cells (HUVEC) were obtained from China Center for Type Culture Collection. HCC cells were cultured in DMEM (Hyclone, Logan city, USA) and HUVECs were cultured in DMEM low-glucose (1,000 mg/L glucose) (Hyclone, Logan city, USA). Cell cultures were supplemented with 10% fetal bovine serum (FBS) and maintained in 5% CO2 humidified atmosphere at 37 °C. DP with purity > 98% was obtained from Ci Yuan Biotechnology Co., Ltd. Shanxi (Xian, China). Insulin-like growth factor 1 (IGF-1) was obtained from PeproTech China (Suzhou, China). DP was dissolved in double distilled water. The HepG2 cells were treated with DP (0, 100, 200, 400 mg/L) for 48 h. Then, conditioned medium (CM) was made from the cell supernatants which collected and filtered by 0.22 µm filter. The collected CM was stored at -80 °C.

### Western Blotting

Protein lysates were prepared, subjected to SDS-PAGE, transferred onto PVDF membranes, and blotted according to standard protocols described previously (Ren et al., 2019). The primary antibodies used in this study were as follows: VEGF (Rabbit mAB, diluted 1:500, Proteintech, USA), HIF-1α (Rabbit mAB, diluted 1:1,000, Abcam, Cambridge, MA, USA), PI3K, phosphor-PI3K (p-PI3K), AKT, phosphor-AKT (p-AKT) (Rabbit mAB, diluted 1:500, Cell Signaling Technology, Beverly, USA), β-actin (Mouse mAB, diluted 1:1,000, Santa Cruz Biotechnology, Santa Cruz, USA).

### Immunofluorescence (IF)

HepG2 and Hepa1-6 cells were cultured on coverslips. Cells were fixed with 4% paraformaldehyde at 4 °C for 15 min and incubated in 0.3% Triton X-100 for 15 min. After blocking with 5% goat serum for 30 min, the cells were incubated with primary antibodies against VEGF (1:200) and HIF-1α (1:200) at 4 °C overnight, and then incubated with Alexa Fluor 488-conjugated or 594-conjugated secondary antibody (Proteintech, USA) for 2 h. DAPI was used to stain nuclear. The immunofluorescent signals were detected by fluorescence microscope (Leica Microsystems, Wetzlar, Germany).

### Cell Counting Kit-8 (CCK8) Assay

1 × 10^3^ HUVECs were seeded to each well of 96-well plates, treated with DP-CM or DMSO (vehicle) for 0, 24, 48, 72, 96, and 120 h, respectively. Then 10μl CCK-8 buffer (Dojindo, Kumamoto, Japan) was added to each well and incubated for 2 h. The absorbance at 450 nm was measured by using Microplate Auto-reader.

### Wound Healing Assay

HUVECs were seeded in 6-well plates and incubated under permissive conditions until 80–90% confluence. Wounds were created in the confluent cells using a sterile 10ul pipette tip. HUVECs were treated with DP-CM or DMSO and images of the scratches were photographed at the identical location of the initial image at 0, 24, and 48 h with inverted microscope (Olympus, Tokyo, Japan). The width of the scratch was analyzed using the Olympus CellSens Dimension software. The assays were performed in triplicate.

### Transwell Migration Assay

Migration of HUVEC were evaluated by a Transwell assay using a 24-well, 8-μm-pore size Transwell plate (Costar, Cambridge, MA). HUVEC (1.5 × 10^5^ cells/well) were seeded in the upper chamber. The lower side of the chamber was filled with CM from the HepG2 cell lines following DP treatments. After 48 h incubation, the migrated cells were stained by 0.1% crystal violet. Migrated cells were photographed by a microscope (Olympus BX51).

### Tube Formation Assay

A 96-well plate was coated with cold Matrigel 50 μl/well and incubated at 37 °C to solidify the Matrigel. HUVECs (4 × 10^4^ cells/well) with different doses (0, 100, 200, 400 mg/L) of DP-treated CM were seeded onto the Matrigel and incubated at 37 °C for 6 h. Tube formation was photographed by a microscope (Olympus, Japan).

### Chick Embryo Chorioallantoic Membrane (CAM) Assay

Fertilized chick eggs were incubated at 37 °C with 70% humidified atmosphere. On the eighth day of incubation, the eggshell was cracked and peeled away from the region over the airspace. 0.5 cm diameter filter papers with DP at different concentrations (0, 100, 200, 400 mg/L, n=6) were placed on the CAMs. The eggs were sealed using sterilized bandages and incubated for another 48 h. The CAMs were photographed with a microscope (Olympus BX51) after fixation (methanol: acetone = 1:1).

### Immunohistochemical Analysis

Balb/c mice were used for tumor growth assay *in vivo*. After subcutaneous injection of Hepa1-6 cells or H22 cells (2×10^6^ cells per mouse) for 6 days, tumor-bearing mice were randomly divided into two groups (n=6): the control group and the DP group. The DP group were intraperitoneally injected with DP at 200 mg/kg once daily for 14 days. The control group was intraperitoneally injected with the same volume of 0.9% normal saline (NS). Dosages and time were determined according to preliminary experiments (Davaatseren et al., 2013; Gulfraz et al., 2014).

Tumor of mice were harvested and embedded in paraffin followed by incubation of target primary antibody at 4 °C overnight. Then these were incubated with HRP conjugated secondary antibodies (Cell Signaling Technology, Beverly, USA) at room temperature for 2 h. The primary antibodies used in this study were as follows: CD31 (Abcam, Cambridge, MA, USA), VEGF (Proteintech, USA), HIF-1α (Abcam, Cambridge, MA, USA). A semi-quantitative evaluation method was applied as follows: the score obtained by the percentage of positive cells (0% = 0; 1–25% = 1, 26–50% = 2, 51–75% = 3, and > 75% = 4) was multiplied by the score obtained by the staining intensity (no staining = 0, weak staining = 1, moderate staining = 2, and strong staining = 3).

### Statistical Analysis

Data were presented as mean ± standard deviation (SD) for normal distribution. Groups were compared by one-way Analysis of variance (ANOVA) and multiple comparisons by LSD-t test by SPSS 21.0 (IBM SPSS for Windows, Version 21.0; IBM Corporation, Armonk, NY, USA). Outliers were excluded with larger or smaller than 2 SD. *P* < 0.05 was considered significant.

## Results

### DP Inhibits Angiogenesis *In Vitro* and *In Vivo*

**Figure 1:**
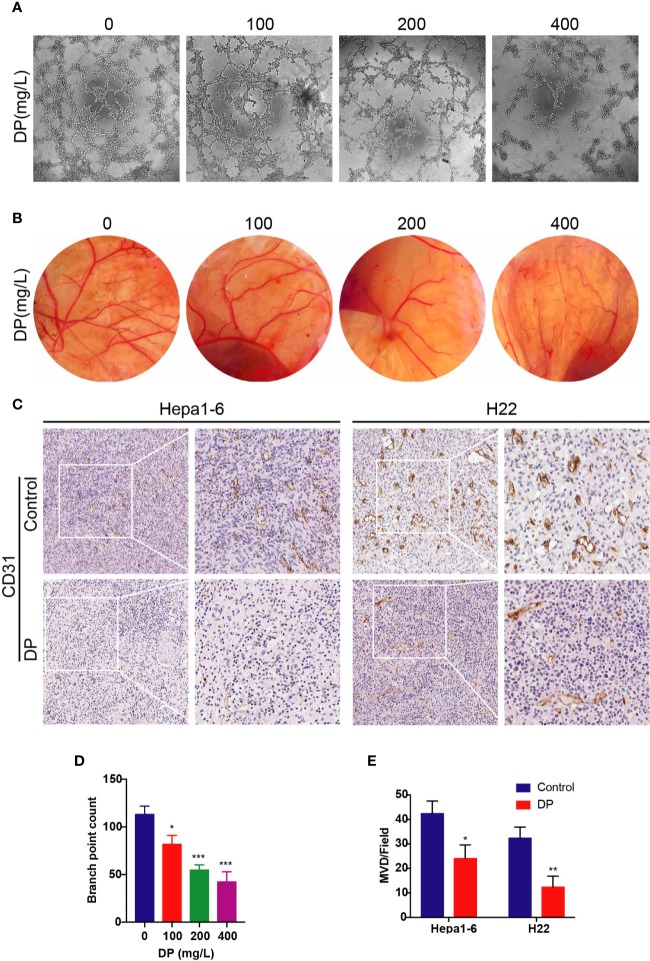
DP inhibits angiogenesis in vivo and in vitro. **(A, D)** Tube formation of HUVECs on Matrigel. Cells were treated with CM derived from 0, 100, 200, and 400 mg/L DP treated HepG2 cells for 48 h. **(B)** The newly formed blood vessels on the chick embryo chorioallantoic membrane (CAM) after treated with DP (0, 100, 200, and 400 mg/L) for 48 h. **(C, E)** Histopathological analyses of tumor growth in mice xenografted with Hepa1-6 and H22 cancer cells. The tumor sections were subjected to IHC staining using an antibody against CD31. The magnification is 400x. Error bars represent mean ± SD from three independent experiments. (*P < 0.05, **P < 0.01, ***P < 0.001).

To assess the antiangiogenic effects of DP, we conducted tube formation assay and chick CAM assay. To get the CM, HepG2 cells were treated with DP (0, 100, 200, 400 mg/L) for 48 h and then the CM was collected. HUVECs were incubated in CM, then tube formation was visualized utilizing microscope photograph technology (**Figures 1A, D**). We observed that CM from DP-treated group significantly prevented HUVECs from forming capillary tubes. After culturing HUVECs with CM derived from 100, 200, and 400 mg/L DP-treatment, the tube formation rate was decreased by 27.73, 51.62, and 62.54% compared to HUVECs cultured with control CM, respectively (*P* < 0.05).

Since we observed that DP inhibited tube formation in HUVECs *in vitro*, we next validated the antiangiogenic effects of DP *in vivo* using CAM assay. After 48 h of incubation, angiogenesis was clearly observed in the fertilized eggs. Importantly, DP treatment caused a reduction of microvessel density in a concentration-dependent manner (**Figure 1B**).

As discussed in the previous study, DP inhibited tumor growth *in vivo* (Ren et al., 2019). To understand whether the inhibition effects of DP contribute to the angiogenesis disruption, IHC of tumor tissues was carried out to evaluate the expression of CD31, a marker representing microvessel density. Hepa1-6 or H22 tumor xenograft mice were randomly divided into two groups: the DP group (treated with DP at 200 mg/kg/d for 14 days) and the vehicle control group (treated with saline for 14 days). Results of IHC revealed that tumors derived from the vehicle control group exhibited markedly higher CD31 expression compared to those from the DP group (**Figures 1C, E**). These results showed that DP inhibited angiogenesis *in vivo* and *in vitro*.

### DP Suppresses HUVECs Migration and Invasion *In Vitro*

**Figure 2:**
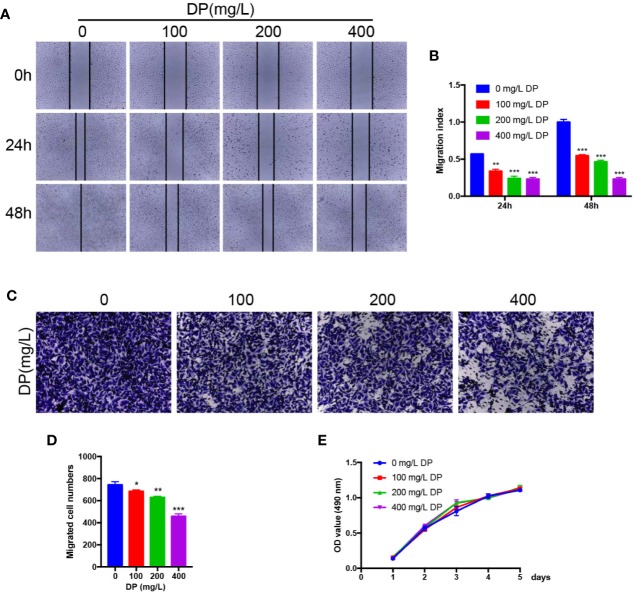
DP suppresses HUVECs migration and invasion *in vitro*. **(A**, **B)** HUVECs were treated with different concentration (0, 100, 200, and 400 mg/L) of DP-CM for 0, 24, and 48 h. Migration ability of HUVECs was evaluated by wound healing assay. **(C**, **D)** HUVECs treated with different concentration (0, 100, 200, and 400 mg/L) of DP-CM were seeded into the inner chamber. Invasion of HMEC-1 cells were evaluated by a Transwell assay. **(E)** The effect of DP-CM on HUVECs proliferation was evaluated by CCK-8 assay. (*P < 0.05, **P < 0.01, ***P < 0.001).

Endothelial cell migration and invasion are crucial steps which involved in angiogenesis. As we showed above, DP suppressed HUVECs capillary tube formation. To explore whether DP influences HUVECs migration and invasion, wound-healing assay, and transwell invasion assay were performed to assess the activity of migration and invasion in HUVECs, respectively. As expected, we observed that various concentrations of DP-treated HepG2 CM significantly decreased the capacity of wound healing in HUVECs compared with those cells without DP-CM treatment (**Figures 2A, B**). We also found that DP-CM markedly inhibited the capacity of HUVECs invasion in a dose-dependent manner in HUVECs (**Figures 2C, D**). Collectively, our data demonstrated that DP suppressed HUVECs migration and invasion.

### DP Treatment Has No Detectable Effect on Proliferation of HUVECs *In Vitro*

To further assess whether DP inhibits the proliferation of HUVECs, we performed CCK8 assay. The HUVECs were subjected to various concentrations of DP-treated CM, and the proliferation rate of the HUVECs was evaluated. Data from CCK8 assay showed that the proliferation rate of HUVECs was comparable between DP-CM treated and vehicle-DM treated HUVECs. (*P* ≥ 0.05) (**Figure 2E**). These data showed that DP had no effect on HUVECs proliferation.

### DP Decreases the Expression of VEGF and HIF-1α in HCC Cells

**Figure 3:**
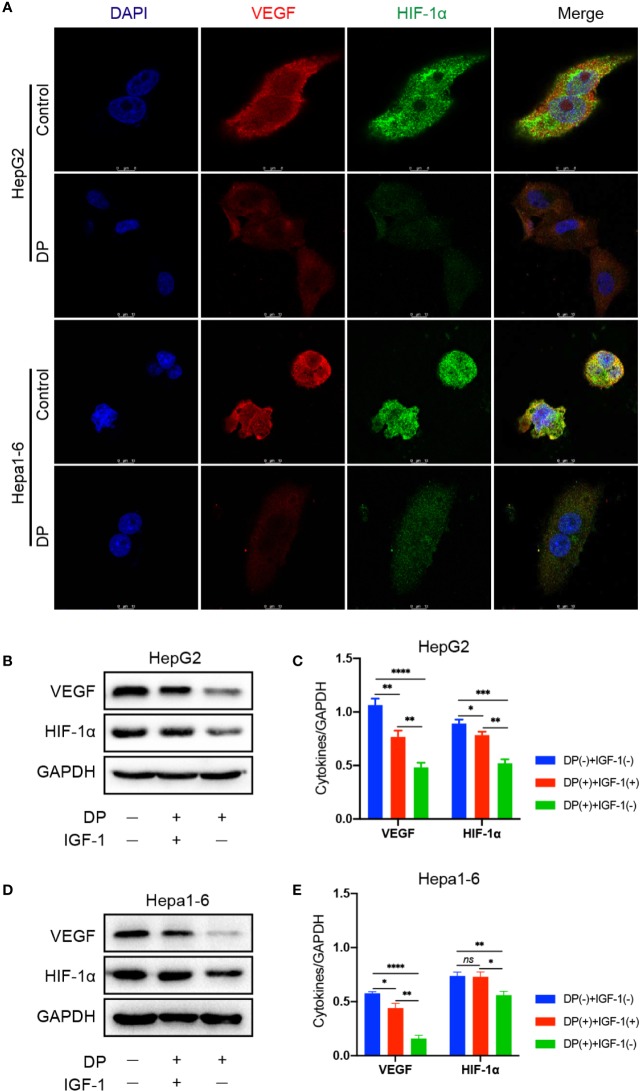
DP decreases the expression of VEGF and HIF-1a in HCC cells. **(A)** Immunofluorescence assays of VEGF and HIF-1a proteins in HepG2 and Hepa1-6 cells treated or untreated with 200 mg/L DP for 48 h. **(B, C)** WB analyses of VEGF and HIF-1a expression in HepG2cells treated or untreated with IGF-1 and DP. **(D, E)** WB analyses of VEGF and HIF-1a expression in Hepa1-6 cells treated or untreated with IGF-1 and DP. No significance (ns), *P < 0.05, **P < 0.01, ***P < 0.001, ****P < 0.0001.

Several studies have shown that VEGF and HIF-1α are important for tumor vasculogenesis. Thus, we assessed the effect of DP on HIF-1α and VEGF expression in HCC cells. The HepG2 and Hepa1-6 cells were selected and treated with 200 mg/L DP or vehicle for 48 h. The expression of VEGF and HIF-1α was evaluated by immunofluorescence staining, respectively. As shown in **Figure 3**, DP treatment significantly decreased the expression of VEGF and HIF-1α. Moreover, the western blot results indicated that the protein levels of VEGF and HIF-1α were markedly downregulated in HCC cells treated with DP as compared to the vehicle treated group.

### DP Decreases the Expression of VEGF and HIF-1α *In Vivo*

**Figure 4:**
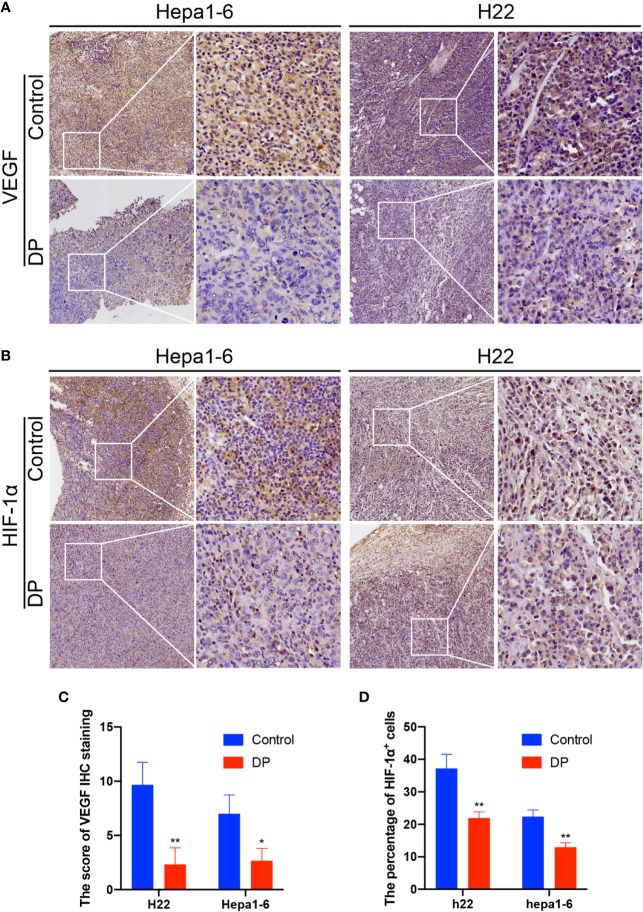
DP reduces VEGF and HIF-1a expression in vivo. Immunohistochemical analysis of tumors from Hepa1-6 and H22 tumor-bearing mice. **(A, C)** Thetumor sections were subjected to IHC staining using an antibody against VEGF. **(B, D)** The tumor sections were subjected to IHC staining using an antibody against IGF-1a. *P < 0.05, **P < 0.01.

Then, we further determined the influence of DP on VEGF and HIF-1α expression *in vivo*. We used the IHC assay to confirm the tumors expression of VEGF and HIF-1α *in situ*. We observed that tumors in the control group exhibited significantly higher VEGF and HIF-1α expression than those of in the DP group (**Figure 4**).

In conclusion, our results demonstrated that the expression of VEGF and HIF-1α could be effectively repressed by DP *in vitro* and *in vivo*.

### DP Regulates VEGF and HIF-1α Expression *via* PI3K/AKT Pathways *In Vitro*

Our previous data demonstrated that DP was an inhibitor for PI3K/AKT/mTOR pathway in HCC cells (Ren et al., 2019). It is well known that PI3K/AKT pathway involved in angiogenesis induced by VEGF. In order to further determine the role of PI3K/AKT pathway plays in DP mediated inhibition of angiogenesis in HCC cells, the expression of key factors involved in the PI3K/AKT signaling pathway was tested in HepG2 and Hepa1-6 cells. The cells treated with 200 mg/L of DP or vehicle for 48 h were harvested and subjected to western blot analysis. The analysis was performed based on the absence or presence of IGF-1, an activator of PI3K/AKT signaling pathway.

**Figure 5:**
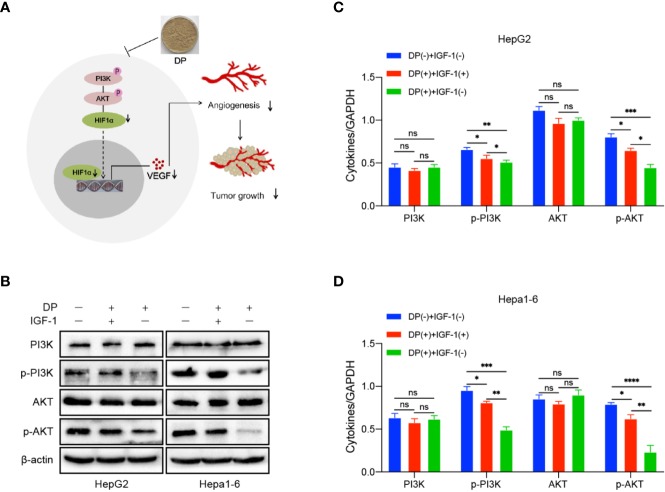
DP inhibits the activation of PI3K/AKT/mTOR pathway. **(A)** WB analyses of p-PI3K, total AKT, p-AKT and total AKT expression in the HepG2 and Hepa1-6 cells treated or untreated with IGF-1 and DP. **(B–D)** Proposed mechanism of DP exerts anti-angiogenesis effect on HepG2 and Hepa1-6 cells. no significance (ns), *P < 0.05, **P < 0.01, ***P < 0.001, ****P < 0.0001.

In HepG2 and Hepa1-6 cells, DP treatment significantly lowered the levels of p-PI3K and p-AKT, which can be reversed with 2 nM IGF-1treatment. Additionally, the decreased expression of VEGF and HIF-1α, resulted by DP treatment, was also recovered by IGF-1 stimulation (**Figures 5A–D**). Therefore, all these results above demonstrated that DP regulated the expression of VEGF and HIF-1α expression through suppression of PI3K/AKT pathways *in vitro*.

### Discussion

Angiogenesis is a vital process involved in tumor growth and metastasis. As HCC is a highly vascularized type of tumor, inhibition of its angiogenesis is considered to be a promising approach with therapeutic value to treat HCC (Nakao et al., 2015; Akhtar et al., 2017; Barbhuiya et al., 2017). The angiogenesis process involves endothelial cell proliferation, invasion, migration, and differentiation into tubular capillaries. In this study, we demonstrated that DP attenuated the process of the tube formation, migration, and invasion of HUVECs *in vitro*. Moreover, DP also significantly decreased the formation of branched blood vessels in CAM, a natural *in vivo* model of angiogenesis. CD31, a molecular marker of microvessel density, was detected by histological analysis of tumor tissues formed from Hepa1-6 and H22 cells. Our data showed that CD31 levels were significantly decreased in tumor tissues following DP treatment. Taken together, these data suggested that DP possessed an ability to inhibit angiogenesis both *in vitro* and *in vivo*.

VEGF, a specific mitogen essential for endothelial cells, has been shown to promote proliferation of endothelial cells, induce angiogenesis, and even enhance the permeability of small vessel. Clinical studies has clarified that the expression of VEGF in HCC is highly related to tumor progression and poor prognosis (Choi et al., 2017; Mao et al., 2017). At the early stage of tumor-induced angiogenesis, a large amount of VEGF is secreted from the cancer cells. The angiogenic effect of VEGF is mainly regulated *via* binding and activating its receptors, e.g.VEGFR2, on endothelial cells (Hida et al., 2016). Previous researches have also revealed that VEGF is a target gene of HIF-1α (a hypoxia response protein). HIF-1α enhanced the transcription level of VEGF, through which to promote neovascularization (Xiao et al., 2015). Our results showed DP administration significantly decreased the expression of VEGF and HIF-1α in HCC cells and subcutaneous tumors, suggesting that DP exerted an inhibitory effect on the tumor angiogenesis *via* blocking the production of VEGF.

PI3K/AKT signaling pathway is an important signaling pathway responsible for malignant cancer cells proliferation, apoptosis, invasion, metastasis, and angiogenesis. Previous studies have shown the PI3K/AKT path way is overactivated in some HCC cases and PI3K/AKT over-activation is associated with poor prognosis in HCC patients (Minguez et al., 2009; Golob-Schwarzl et al., 2017). It has been revealed that PI3K/AKT activation enhances the transcriptional activity of HIF-1α, further inducing VEGF expression (Zhang et al., 2015). We previously reported that DP exhibited significant inhibitory eﬀects on the growth of HCC cells *via* attenuating PI3K/AKT signaling pathway (Ren et al., 2019). In the present study, our results showed that DP markedly decreased the protein levels of p-PI3K and p-AKT in HCC cells, suggesting that DP is capable of modulating PI3K/AKT signaling. Notably, pre-treatment of HCC cells with IGF-1 significantly recovered the protein levels of VEGF, HIF-1α, p-PI3K, and p-AKT which were downregulated by DP treatment. In contrast, neither DP nor IGF-1 treatment obviously changed the protein levels of PI3K and AKT. In conclusion, we have demonstrated that DP displays a remarkable antiangiogenic effect both *in vivo* and *in vitro* and the mechanism appears to be associated with the inhibition of VEGF and HIF-1α expression by targeting PI3K/AKT pathway.

Taken together, the current study extended our understanding on the molecular mechanisms of DP on anti-angiogenic activity in HCC cancer cells. DP could be deemed as a promising tumor angiogenesis inhibitor and can be developed as a drug candidate in clinical treatment in the future. In this study, the *in vivo* model using xenografted Hepa1-6 and H22 cells in mice may not completely correspond to natural growth of tumors in patients. Therefore, immunodeficient nude mice and human HCC cells will be used for the experiments in the further.

## Data Availability Statement

All datasets generated for this study are included in the article/supplementary material.

## Ethics Statement

The animal study was done according to the Principle of Laboratory Animal Care (NIH Publication No. 85-23, revised 1985). The protocol was approved by the Animal Ethics Committee of Xinxiang Medical University.

## Author Contributions

FR and JL designed the experiments, and FR wrote the manuscript. JL, KW, YuY, YiY, and YW carried out the experiments and analyzed the data. FR supervised and corrected the manuscript.

## Funding

This work was supported by Key Science and Technology Program of Henan [182102310259 and 192102310097]; Young Teachers Training Projects of Universities in Henan province [2018GGJS103]; Key Scientific Research Project of Higher Education of Henan Province [19A310004]; Doctoral research funding of Xinxiang Medical University [XYBSKYZZ201646]; Henan Provincial Medical Science and Technology Research Project [SB201901064].

## Conflict of Interest

The authors declare that the research was conducted in the absence of any commercial or financial relationships that could be construed as a potential conflict of interest.
